# Using of prothrombin complex concentrate in patients with trauma-induced coagulopathy

**DOI:** 10.1186/2197-425X-3-S1-A908

**Published:** 2015-10-01

**Authors:** O Tarabrin, O Chystikov, S Shcherbakov, D Gavrychenko, G Mazurenko, P Tarabrin

**Affiliations:** Odessa National Medical University, Anesthesiology, Intensive Care with Postgraduate Education, Odessa, Ukraine; Odessa National Medical University, Odessa, Ukraine

## Introduction

The relevance of trauma-induced coagulopathy is a major factor leading to a fourfold increase in the mortality in patients with polytrauma (K. Brohi et al., 2007). The main reason associated with traumatic injury deaths is uncontrolled bleeding.

## Objectives

To compare the effectiveness of prothrombin complex concentrate (PCC) and fresh frozen plasma (FFP) in patients with multiple trauma, complicated with coagulopathy bleeding.

## Methods

The study involved 63 patients who entered the Odessa Regional Hospital with traumatic injuries: concomitant skeletal trauma, fractures of femur and humerus. Patients were divided into 2 groups: 1^st^ group (n = 32) as a treatment of coagulopathy was received PCC in a dose of 1 ml/kg (25 IU/kg) at time of admission to the intensive care unit (ICU); 2^nd^ group (n = 31) received FFP in a dose of 15 ml/kg. Evaluation of the functional state of the hemostasis system was carried out using low-frequency piezoelectric thromboelastography (LPTEG) on admission to hospital and 24 hours after the patient's admission to the ICU.

## Results

According to LPTEG indicators polytrauma patients has a statistically significant abnormalities in all parts of hemostatic system: platelet aggregation - Intensity of contact coagulation (ICC), the coagulation - Intensity of coagulation drive (ICD), clot maximum density (MA) and fibrinolytic activity - Index of retraction and clot lysis (IRCL). ICC in patients with multiple injuries was reduced by 28.49%, ICD was less than normal at 35.78%, MA was reduced by 76.67%, IRCL was 91,16% above the norm. Patients of 1^st^ group according LFTEG had significant changes in all parts of coagulation 24 hours after the intensive care. Indicators of platelet hemostasis characterized by persistence of hypoagregation: ICC was reduced by 24.71%, compared to the norm; parameters of coagulation and fibrinolysis have reliable trend toward normal and decreasing the activity of fibrinolysis index reaches normal reference values. Patients of 2^nd^ group have hypoagregation and hypocoagulation state with increased active of fibrinolysis: ICC was reduced by 24.72%, ICD reduced by 20.76%, MA was reduced by 23.54%, IRCL was above the norm to 25.21%. Clinically, patients of the 1^st^ group had reducing the volume of blood transfusions as opposed to the 2^nd^ group.

## Conclusions

Patients with multiple injuries have violation in all parts of hemostatic system. The use of prothrombin complex concentrate can reduce the severity of pathological changes in the hemostasis in patients with polytrauma.

